# Reversible switching between pressure-induced amorphization and thermal-driven recrystallization in VO_2_(B) nanosheets

**DOI:** 10.1038/ncomms12214

**Published:** 2016-07-18

**Authors:** Yonggang Wang, Jinlong Zhu, Wenge Yang, Ting Wen, Michael Pravica, Zhenxian Liu, Mingqiang Hou, Yingwei Fei, Lei Kang, Zheshuai Lin, Changqing Jin, Yusheng Zhao

**Affiliations:** 1 High Pressure Science and Engineering Center, University of Nevada, Las Vegas, Nevada 89154, USA; 2 HPSynC, Geophysical Laboratory, Carnegie Institution of Washington, Argonne, Illinois 60439, USA; 3 Center for High Pressure Science and Technology Advanced Research (HPSTAR), Pudong, Shanghai 201203, China; 4 Institute of Nanostructured Functional Materials, Huanghe Science and Technology College, Zhengzhou, Henan 450006, China; 5 Geophysical Laboratory, Carnegie Institution of Washington, Washington, District of Columbia 20015, USA; 6 Beijing Centre for Crystal Research and Development, Technical Institute of Physics and Chemistry, Chinese Academy of Sciences, Beijing 100190, China; 7 National Laboratory for Condensed Matter Physics, Institute of Physics, CAS, Beijing 100190, China; 8 Southern University of Science and Technology, Shenzhen 518055, China

## Abstract

Pressure-induced amorphization (PIA) and thermal-driven recrystallization have been observed in many crystalline materials. However, controllable switching between PIA and a metastable phase has not been described yet, due to the challenge to establish feasible switching methods to control the pressure and temperature precisely. Here, we demonstrate a reversible switching between PIA and thermally-driven recrystallization of VO_2_(B) nanosheets. Comprehensive *in situ* experiments are performed to establish the precise conditions of the reversible phase transformations, which are normally hindered but occur with stimuli beyond the energy barrier. Spectral evidence and theoretical calculations reveal the pressure–structure relationship and the role of flexible VO_*x*_ polyhedra in the structural switching process. Anomalous resistivity evolution and the participation of spin in the reversible phase transition are observed for the first time. Our findings have significant implications for the design of phase switching devices and the exploration of hidden amorphous materials.

## Abstract (web summary)

Pressure can either make materials more disordered, like amorphization, or more thermodynamic stable, yet the switching between the two metastable phases has not been described. Wang *et al*. study it in vanadium oxide nanosheets and highlight the role played by spin and charge during the transition.

## Graphical abstract

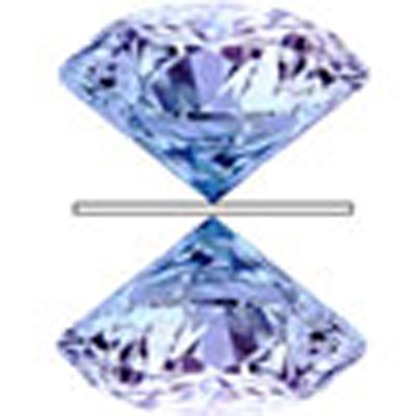

Materials that contain no long-range structural order (for example, glass or amorphous phase) are fundamentally interesting to both the basic sciences and for practical industrial applications^1^,^2^. Glass, which has been considered a metastable and kinetically frozen disordered state, can be synthesized by rapidly quenching a melt from high temperature to prevent crystallization. Another frequently reported but distinct approach to amorphous phases or glasses is via pressure-induced amorphization (PIA)^3^,^4^. The disordering process in PIA differs significantly from the thermally-induced disorder in substance melting, where a much higher density is expected. PIA has been observed in many materials such as ice^5^,^6^,^7^, α-quartz SiO_2_ (refs 8, 9, 10), AlPO_4_ (ref. 11), R-Al_5_Li_3_Cu (ref. 12), and so on, and is widely accepted as an important condensed matter phenomenon. In some cases, mechanical instability caused by ‘thermodynamic melting' and increased atomic coordination are considered to contribute most to PIA^8^,^13^. While in other cases, such as ZrW_2_O_8_, negative thermal expansion (NTE) may have some possible connection with the PIA^14^,^15^,^16^,^17^. However, determining the underlying mechanism still remains one of the most fascinating challenges and some recent evidence even shows that previously reported PIAs were due to the fragmentation of bulky particles into nanocrystals and strongly correlated pressurization environments^3^. Furthermore, new high-density PIA generated materials are expected to drive new theoretical approaches for modelling this phenomenon, which is essential for discovering their practical applications. Recently, pressure-induced crystallization revealed the topological ordering in metallic glasses at room temperature^18^. But upon heating, the recovered amorphous phase tends to return to its stable crystalline phase to minimize the system's energy, depending on the kinetic barrier it needs to overcome. Such examples include the main group compounds SnI_4_, LiKSO_4_, Ca(OH)_2_, clathrasils and berlinite AlPO_4_ (refs 19, 20, 21, 22, 23). Molecular dynamics calculations on PIA-AlPO_4_ (ref. 23) showed that the presence of non-deformable, fourfold coordinated PO_4_ interlinking units in the crystal structure have a crucial role in the reversible amorphization and crystallization phase transition, where they acted as templates around which the original structure and even the original orientation were restored^23^,^24^. So far, reversible PIA has only been observed in the above-mentioned main group compounds, where only the lattice participates in the intriguing structure transformation. The study of more variables (that is, charge, spin and orbital) involved in PIA and the subsequent recrystallization processes could potentially allow a more comprehensive understanding of the order–disorder transformation mechanism and the electronic behaviour in highly disordered materials.

Discovering new external-stimuli responsive compounds with switchable ground states is a major objective in material science because these materials often lead to unusual phenomena or useful functionalities^25^,^26^. When considering PIA materials as metastable and dense phases distinct from their crystalline forms, it is important to achieve a controllable phase switching between either the PIA glass and their crystalline polymorphisms or even between two PIA phases.

In this work, we report a reversible structural switch between the metastable crystalline phase VO_2_(B) and its PIA glass, in the form of nanosheets. The phase switch between these two metastable phases is realized with low-pressure compression (∼20 GPa) and relative low-temperature annealing (∼200 °C). The phase transformation and underlying mechanism is thoroughly studied using *in situ* synchrotron X-ray diffraction (XRD), infrared, Raman spectroscopy and theoretical calculations. High-pressure electronic transport properties and magnetic properties are also studied to provide direct evidence of the participation of charge and spin during the phase transformation, for the first time. Our findings may provide a new research platform for the exploration of novel amorphous materials and phenomena under controllable external stimuli.

## Results

### Material and crystal structure

**Figure 1:**
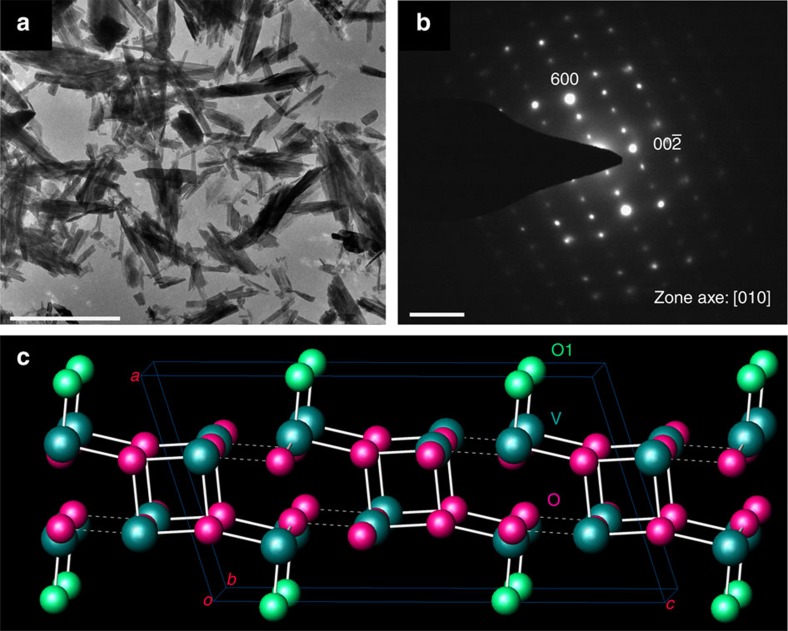
TEM characterizations and crystal structure of VO_2_(B) at ambient pressure. (**a**) Bright field TEM images of VO_2_(B) nanosheets. Scale bar, 2 μm. (**b**) Selected-area electron diffraction of VO_2_(B) nanosheets along the [010] zone axis showing its single crystal nature. Scale bar, 5 nm^−1^. (**c**) Crystal structure of VO_2_(B) showing a dense packed vanadium–oxygen layer in the *bc* plane and *ac* plane with hierarchical V–O bonding. The O1 atoms with shortest V=O bonds are highlighted in green.

There are several crystalline phases of VO_2_ at ambient conditions: VO_2_(A), VO_2_(B), VO_2_(M), VO_2_(R) and other metastable phases^27^,^28^,^29^,^30^,^31^. Among them, VO_2_(M) is the thermodynamically stable phase but it undergoes an insulator-to-metal transition (ITM) under high pressure to a metallic phase VO_2_(R) around room temperature. The structures and physical properties of the phases differ significantly due to the distinct coordination environments of V atoms in the VO_*x*_ polyhedra, the V^4+^–V^4+^ interactions and the various cross linking manners (Supplementary Fig. 1). VO_2_(B) is a thermodynamically metastable phase adopting an anisotropic layered structure, and is frequently used as a battery material^32^. Its unique structural features, such as well-embedded layers and hierarchical V–O bonding, also make VO_2_(B) an interesting candidate for structural stability studies under high pressure. In this work, VO_2_(B) nanosheets were synthesized from high pure V_2_O_5_ raw material via a hydrothermal route with citric acid as the reducing agent^33^. The product consisted of well-shaped nanosheets that were several micrometres in length/width and tens of nanometres in thickness, and its single crystal nature is proven by the well-arranged spots in the selected-area electron diffraction pattern (Fig. 1a,b). The structure of VO_2_(B) is characterized by the layered feature in the *bc* plane with a twofold connected O1 atom between the layers (Fig. 1c). Less electron charge distribution between the layers makes the [100] direction more compressible, and there is also a distinct gap along the [001] direction. Overall, the VO_2_(B) structure has a hierarchical structure consisting of condensed face-sharing VO_*x*_ polyhedra. More structural features of VO_2_(B) along different axes are shown in Supplementary Fig. 2.

### PIA at room temperature

**Figure 2:**
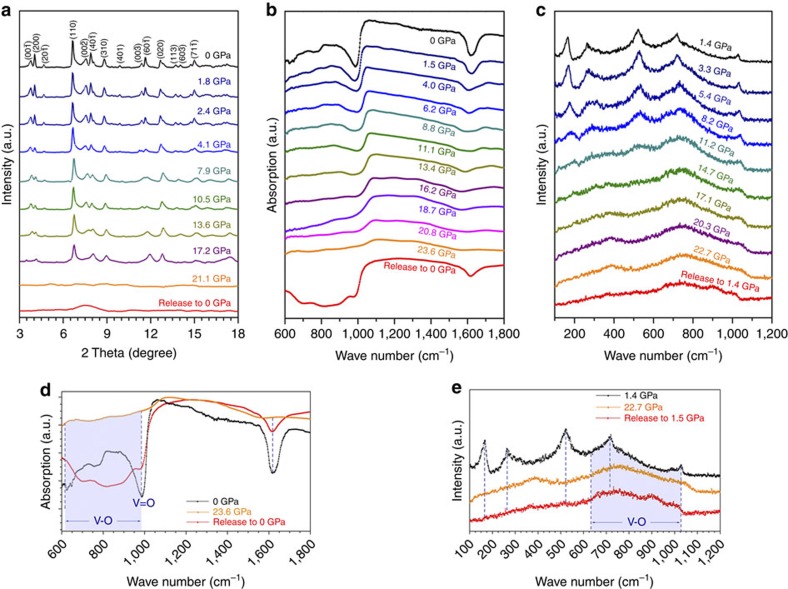
Diffraction and spectral evidences for VO_2_(B) nanosheets under compression. (**a**) *In situ* synchrotron XRD patterns of VO_2_(B) as a function of pressure show the pressure-induced amorphization happened around 21 GPa. (**b**) Infrared spectra taken between ambient pressure and 23.6 GPa. (**c**) Raman spectra taken between 1.4 and 22.7 GPa. (**d**,**e**) Detailed infrared and Raman spectra at the starting, highest pressure and recovered states.

The *in situ* synchrotron XRD patterns of VO_2_(B) nanosheets, as a function of pressure without pressure transition medium, are shown in Fig. 2a. There was no structural transition until the onset of amorphization around 20 GPa. The XRD pattern at ambient conditions is well-indexed with the monoclinic space group *C*2/*m* and lattice constants *a*=12.054(3) Å, *b*=3.693(1) Å, *c*=6.424(2) Å and *β*=106.96(1)°, in good agreement with other reported values^12^,^13^. As discussed above, from a structural chemistry viewpoint, the VO_2_(B) nanosheet was expected to show an anisotropic compressibility under pressure. The lattice parameters at various pressures before PIA were obtained by Reitveld refinements of these XRD patterns, as shown in Supplementary Fig. 3. During compression, the diffraction peaks became broader and weaker from about 17 GPa, and completely vanished around 20 GPa, indicating the loss of long-range ordering in the pressure-amorphized state. To check the shear and non-hydrostaticity effect on the PIA, we repeated the experiments using neon and helium as pressure-transmitting medium (PTM) for comparison. In all cases, the PIA process occurred and the amorphous PIA-VO_2_(B) was preserved to ambient conditions after releasing pressure. In contrast, no PIA was observed in the thermal-stable VO_2_(M) phase up to 55 GPa^34^.

Spectroscopic techniques can probe the short-range structural features of local atomic coordinates. We employed infrared and Raman spectroscopy to examine the local structural evolution of VO_2_(B) nanosheets during compression and decompression. Figure 2b displays the infrared spectrum of VO_2_(B) as a function of pressure up to 23.6 GPa. Characteristic peaks located around 1,000 cm^−1^ (at ambient conditions) are assigned to the shortest V^4+^=O1 bonds pointing perpendicularly into the *bc* interlayer. The mode frequency barely shifted (<50 cm^−1^) towards high wave numbers upon compression. However, the broadening of the vibrational mode increases with pressure, and finally merging with other broad bands down to 600 cm^−1^ is a more profound change. These were associated with angle deformations of the VO_*x*_ polyhedral upon compression and eventually a disordered state resulted in amorphization. This evidence clearly indicates the nature of the degenerated chemical environments of oxygen around the V^4+^ centres. As pressure increases, the enhanced oxygen coordination around vanadium atoms in VO_2_(B) is expected to lead to the dynamical lattice instability, which triggers the PIA. It is interesting that the preserved PIA-VO_2_(B) has similar local vibrational modes to the pristine VO_2_(B) nanosheets, indicating that the short-range structure features of VO_2_(B) are preserved, as indicated in Fig. 2d. Moreover, Raman spectroscopy was employed to evaluate the contribution of the electron–phonon interactions to the PIA phase transition of VO_2_(B) (Fig. 2c). At low pressure, only five bands near 198, 260, 570, 780 and 1,020 cm^−1^ were observed with moderate intensity in the VO_2_(B) nanosheets. Typically, the peak located at 1,020 cm^−1^ can be assigned to the V^4+^=O1 stretching modes of terminal oxygen atoms. Upon compression above 10 GPa, all the bands weakened and finally vanished. Meanwhile, broad bands between 600 and 1,000 cm^−1^ were observed corresponding with the PIA process. As anticipated, similarly to the infrared results, all of the atomic location information was preserved with a more disordered state as indicated by the broadening of the vibrational bands in Fig. 2e, which closely resembles the Raman changes during the ITM process from VO_2_(M) to metallic VO_2_(R).

### Thermal-driven recrystallization and the phase diagram

**Figure 3:**
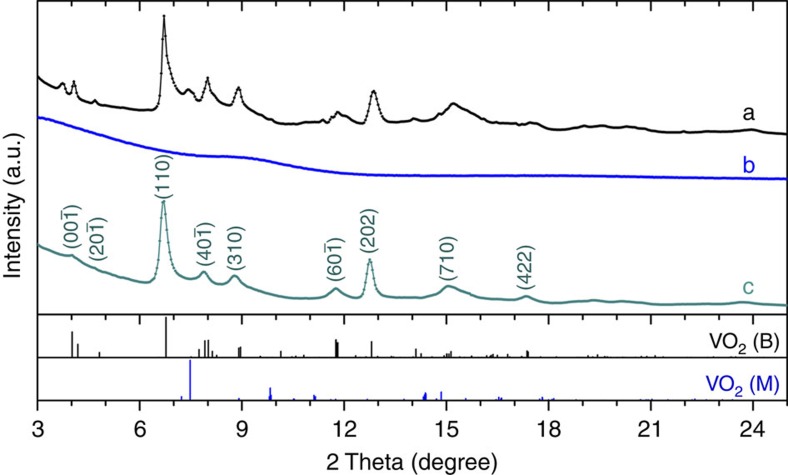
XRD patterns of VO_2_(B) nanosheets upon compression and heating. (**a**) XRD pattern of the pristine VO_2_(B) nanosheets; (**b**) XRD pattern of PIA-VO_2_(B) recovered from 31.5 GPa; (**c**) XRD pattern of PIA-VO_2_(B) after annealing at 250 °C in a vacuum for 5 min. Bottom: theoretical Bragg lines of VO_2_(B) and VO_2_(M). The peaks of (**c**) are indexed with the monoclinic space group *C*2/*m* and lattice constants: *a*=12.065(3) Å, *b*=3.650(9) Å, *c*=6.482(11) Å and *β*=107.53(7)°.

The PIA-VO_2_(B) phase returns to the pristine VO_2_(B) structure upon annealing at relatively low temperature (∼200 °C by *in situ* annealing experiment) for a short time (∼5 min). Figure 3 shows the XRD pattern of the starting VO_2_(B) nanosheets and PIA-VO_2_(B) before and after annealing at 250 °C (50 °C above the critical temperature to guarantee a complete recrystallization). To ensure that the PIA process was achieved, a higher pressure of 31.5 GPa (far beyond the PIA starting point) was applied. As discussed, the loss of the X-ray diffraction peaks in the recovered PIA-VO_2_(B) sample suggests the absence of long-range ordering. After annealing at 250 °C in a vacuum environment for 5 min, surprisingly, we noticed that the diffraction pattern from this heat-treated sample showed the same powder characteristic peaks as the VO_2_(B) phase with a monoclinic space group *C*2/*m*, lattice constants *a*=12.065(3) Å, *b*=3.650(9) Å, *c*=6.482(11) Å and *β*=107.53(7)° (Supplementary Fig. 4) and a little expanded cell volume (273.5 Å^3^ versus 272.2 Å^3^ of the pristine sample). This structure switching phenomenon is distinct from the previously reported reversible PIA phenomena with the following characteristics:^23^,^24^ (**1**) In the cases of some so-called ‘memory glasses', such as AlPO_4_, the PIA phases can be restored to their initial crystalline structures spontaneously at room temperature once pressure is released. In contrast, PIA-VO_2_(B) can exist as a metastable, high density, intermediate phase, and the small active energy barrier between PIA-VO_2_(B) and crystalline VO_2_(B) enables feasible control of the phase switch; (**2**) The reversible PIA and recrystallization processes are guaranteed to occur between VO_2_(B) and PIA-VO_2_(B) (except some thermodynamically stable phases such as VO_2_(M)), due to the relatively low operating temperature (compression at room temperature and annealing at ∼200 °C). The illustration that dynamically low temperature can hinder both the pressure- and temperature-induced traditional phase transitions indicates that more hidden phase relationships may be discovered at low temperature. (**3**) Moreover, the crystal structure of VO_2_(B) is built up of a 3*d* metal V^4+^ (*S*=1/2) based framework. Both the atomic coordination preference of the distorted VO_*x*_ polyhedra and possible electron/magnetic interactions within the crystal lattice may contribute to the reversible structure transformation. The latter will be discussed in detail in the electron transport property section.

**Figure 4:**
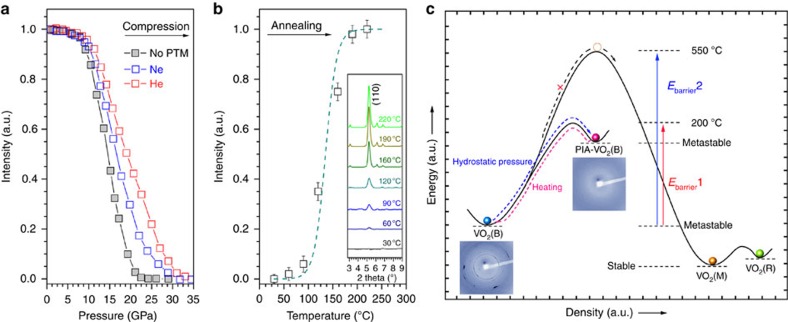
Diffraction intensity and phase scheme of VO_2_. (**a**) The integrated (110) peak intensity of VO_2_(B) as a function of pressure under three different compression conditions. (**b**) The integrated (110) peak intensity of PIA-VO_2_(B) as a function of the annealing temperature. The error bars in (**b**) indicate the s.d. values of the integrated peak intensity at a given pressure. (**c**) Density-energy scheme of the VO_2_ system and the reversible structure switching. Blue and red arrows show the thermal dynamically allowed phase transitions between VO_2_(B) and PIA-VO_2_(B); The black arrow shows the dynamically hindered transition from VO_2_(B) to VO_2_(M) under compression.

Recent investigations by more powerful and precise techniques show that PIA is highly related to the pressurization environments and crystallinity of the starting materials. Some of the phenomena previously reported as PIA were likely due to either the formation of multiple polymorphic phases, or even that the PIA process did not occur if single crystal samples were adopted or more hydrostatic compression was applied^8^,^9^,^10^,^35^. To check this issue in the VO_2_(B) system and obtain the exact conditions where the phase transformations occurred, *in situ* powder XRD experiments were conducted. Firstly, the PIA process of VO_2_(B) with different PTMs were evaluated. Figure 4a displays the integrated (110) peak intensity of VO_2_(B) as a function of pressure without a PTM or with Ne, He as the PTMs. VO_2_(B) nanosheets under all of these three conditions showed the onset of PIA around 10 GPa. Without PTM, the PIA process accomplished concluded around 20 GPa or a little higher when a better hydrostatic pressure condition was given (30 and 35 GPa for neon and helium as PTMs, respectively). This indicates that the PIA of VO_2_(B) is somewhat related to the deviatoric stress^36^, which is reasonable considering the hierarchical structural features of VO_2_(B). Fortunately, the value of 20 GPa was low enough to obtain bulk samples using a large volume press apparatus for routine magnetism measurements. Figure 4b shows the (110) peak intensity of PIA-VO_2_(B) as a function of the annealing temperature. The recrystallization starts from as low as 100 °C and completes around 200 °C. The relatively low annealing temperature makes the switch between PIA-VO_2_(B) and crystalline VO_2_(B) feasible. The pressure- and temperature-induced phase transformations of VO_2_(B) nanosheets observed in our work are summarized schematically in Fig. 4c with other known VO_2_ crystalline phases. Under compression at room temperature, the expected phase transformation from metastable VO_2_(B) to thermodynamically stable VO_2_(M) does not occur. This indicates a higher kinetic barrier (*E*_barrier_2) of atomic diffusion, and the large surface energy from the nanosheets at room temperature. The formed high-density PIA-VO_2_(B) returned to the pristine metastable VO_2_(B) structure after short-time annealing at relatively low temperature (250–300 °C, 10 min), instead of transforming to the thermodynamically stable VO_2_(M) phase. This indicates a relatively low kinetic barrier transforming to metastable VO_2_(B) compared with the high crystallization energy associated with the VO_2_(M) phase and demonstrate that hidden structural switching can be realized using a compression and heating route, as shown in Fig. 4c. Such an intriguing phase transition is the first ever example observed in a 3*d* metal involved material. Similar structure switching behaviour was observed in PbTe nanoparticles^37^, but in our case the switching between two thermally metastable phases was highly dependent on the temperature parameter. We believe that the key factor enabling this reversible phase transformation is the temperature, which is kinetically low enough for sufficient atomic mobility, which thus causes the elastic deformation of the structure. However, when the annealing temperature was high enough to surpass the VO_2_(B) to VO_2_(M) phase transition temperature (∼550 °C)^33^, the PIA-VO_2_(B) can also transfer to VO_2_(M) via the intermediate VO_2_(B) phase.

### First-principles calculation of the pressure-phase relationship

**Figure 5:**
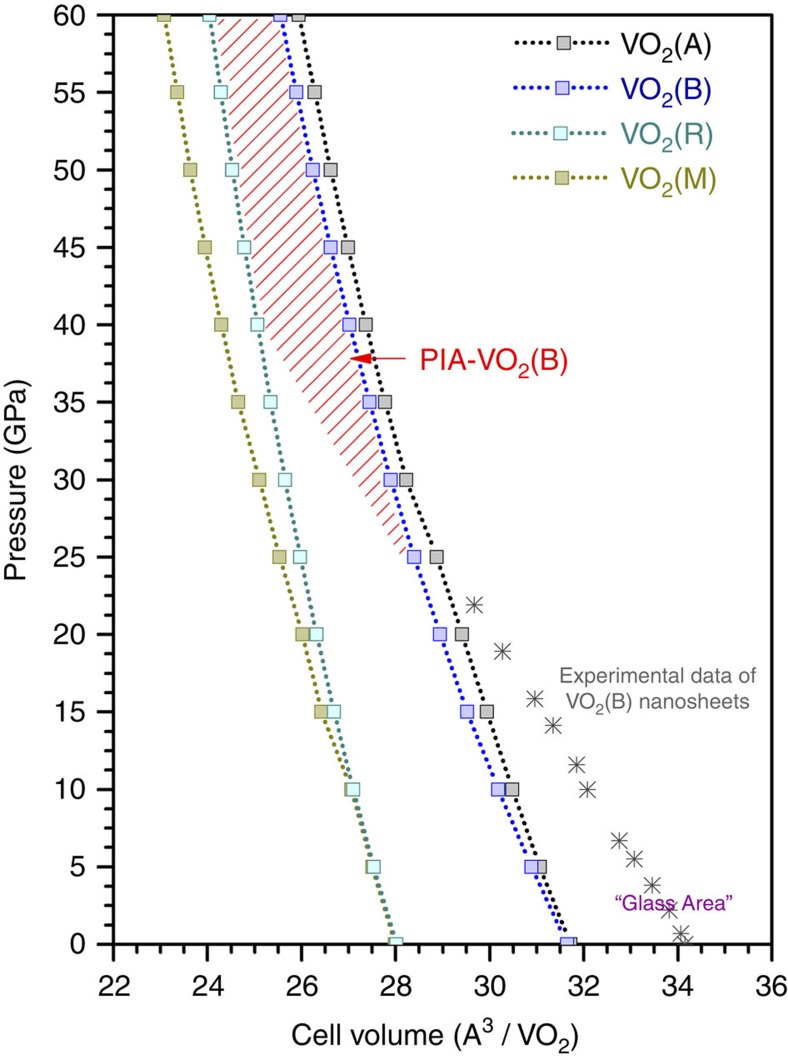
Pressure–volume equations of state of VO_2_ phases. Squares present the simulated results based on DFT calculations of VO_2_(A), VO_2_(B), VO_2_(M) and VO_2_(R) upon compression up to 60 GPa; asterisks are the experimental data of the VO_2_(B) nanosheets. The equation of state of VO_2_(B) nanosheets has the parameters: zero-pressure volume *V*_0_=34.325 Å^3^ per VO_2_, bulk modulus *B*_0_=129.1 GPa and bulk modulus pressure derivative *B*_0_′=4.

The experimental results evidently show the pressure- and thermal-controlled structure switching of VO_2_(B) nanosheets. To provide further thermodynamic and kinetic insight into the pressure-phase relationship during the phase transformation, we performed density functional theory (DFT) simulations using CASTEP code^38^. Detailed calculation information is provided in the Supplementary Materials. Total energy calculations demonstrate that VO_2_(M) is the most stable phase of VO_2_ under ambient pressure at room temperature, and that VO_2_(M) and VO_2_(R) have a higher density than VO_2_(A) and VO_2_(B) (Supplementary Fig. 5). Upon compression up to 100 GPa, VO_2_(B) was thermodynamically metastable compared with VO_2_(M) and VO_2_(R). However, no crystal-to-crystal phase transition from VO_2_(B) to VO_2_(M) or VO_2_(R) was observed, due to the high-kinetic energy barrier between them at room temperature. Figure 5 shows both the calculated pressure–volume equations of states of the four crystalline VO_2_ phases and the experimental data of VO_2_(B) nanosheets upon compression. The experimental data of the VO_2_(B) nanosheets exhibit a larger unit cell volume than those theoretically predicted, which is reasonable considering the nanosheet surface defects. This large cell volume falls into the traditional VO_2_ glass region. Upon compressing to around 20 GPa, the VO_2_(B) nanosheets passed through the ideal pressure–volume boundary of VO_2_(B) and became amorphous with a higher density. The stable region of PIA-VO_2_(B) is indicated in Fig. 5, which passed between VO_2_(B) and VO_2_(M) under high pressure. Several possibilities have been proposed for the underlying mechanism of a PIA process, such as the mechanical instability beyond the Born stability conditions and ‘thermodynamic melting'^1^,^9^. In the case of VO_2_(B), we propose that the combination of kinetic hindrance of the phase transformation to thermodynamically stable VO_2_(M) and the increased atomic coordination are the driving forces of PIA. Upon moderate temperature annealing, the preference of VO_*x*_ polyhedra in thermodynamically metastable PIA-VO_2_(B) drives the structure to return to the pristine VO_2_(B).

In the P–V phase diagram, one can clearly see the difference between PIA-VO_2_(B) and VO_2_ glass. It is important to note that other hidden phases of VO_2_ may exist, either in the low-density glass range or the high-density pressure-induced amorphous range. In this study, we observed profound structure switching behaviour of PIA-VO_2_(B) at kinetically moderate temperatures, which demonstrates an interesting phenomenon of how temperature has a critical role in governing phase transformations under high pressure. The exploration of yet more interesting physical properties related to high density VO_2_ glass with a short-range order may greatly boost the development of condensed matter science.

## Discussion

**Figure 6:**
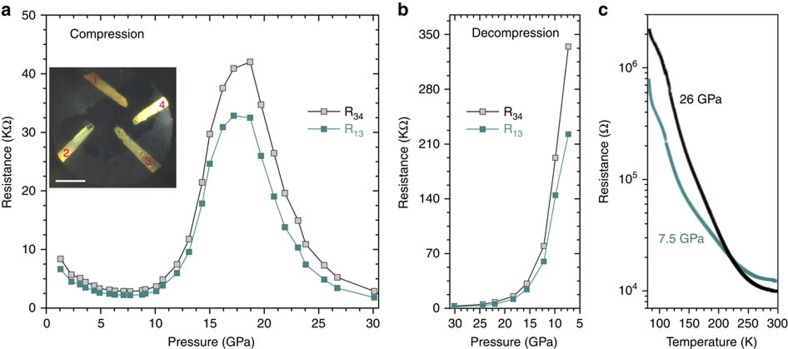
Electrical resistances of VO_2_(B) nanosheets as a function of pressure. (**a**) Compression. Scale bar, 100 μm. (**b**) Decompression. (**c**) Temperature dependence of the sample before and after the pressure-induced amorphization. The inset in (**a**) shows the microphotograph of the VO_2_(B) sample inside a DAC with four Au probes.

VO_2_ phases as the famous strongly correlated family always shows interesting magnetic and electric properties. To gain more insight into the participation of charge and spin interactions during the PIA, *in situ* electrical resistance within a diamond anvil cell (DAC) and *ex situ* magnetic susceptibility measurements (using an amorphized sample synthesized by a large volume press apparatus) were performed. Figure 6 shows the resistance change during compression and decompression, and those at 7.5 and 26 GPa (before and after amorphization) as a function of temperature. At the first stage (*P*<10 GPa), the resistance drop can be associated with the broadening and partial overlapping of the valence and conduction bands, caused by the normal pressure-induced shortening of V–V distances and bending of the V–O bonds. A steep increase in the resistance, which is caused by the onset and completion of the PIA, dominates the profile between 10 and 18 GPa. During the PIA process, point defects accompany the breaking of the long-range order, and thus the electron transport is suppressed by these increased defects, which serve as scattering centres. After the PIA process, band overlapping proceeds, as revealed by the continuous decrease of the electrical resistance, and finally drops to 30 GPa; nearly the same level as the crystalline VO_2_(B) before PIA. However, after decompression, the resistance increases by two orders of magnitude than the pristine VO_2_(B) sample (Fig. 6b), which indicates a reinforced electronic localization in PIA-VO_2_(B). The temperature dependence of the electric resistance before and after the PIA process (7.5 and 26.0 GPa) proves that PIA-VO_2_(B) remains a semiconductor but has poorer electrical conductivity (Fig. 6c). We also fabricated PIA-VO_2_(B) samples using a large volume press apparatus at room temperature and 20 GPa. The decrease of the effective Bohr magnetic moment in PIA-VO_2_(B) from 1.35*μ*_B_ to 1.15*μ*_B_ per V^4+^, as derived from the *ex situ* magnetic susceptibility measurement, suggests that the electron localization may be due to the formation of more V–V pairs/dimers (Supplementary Fig. 6). More investigations are required to further probe the role of charge and spin in the PIA phenomenon and structure memory of VO_2_(B) nanosheets.

In conclusion, we report a precise control of the structure switching between crystalline and pressure-induced amorphous phases in VO_2_(B) nanosheets for the first time. The PIA-VO_2_(B) has no long-range periodicity, but preserves the inherited VO_*x*_ polyhedral from VO_2_(B) with highly distorted local ordering. Upon moderate temperature annealing, these VO_*x*_ polyhedra release excess energy and restore the pristine long-range periodicity. Spectral evidence and DFT calculations provide additional insight into the phase relationship between crystalline VO_2_ phases and PIA-VO_2_(B) glass. The dynamically moderate annealing temperature provides a key mechanism of energetic control on the hidden reversible amorphization of metastable VO_2_(B). Preliminary evidence for the participation of charge and spin interactions during PIA were also observed for the first time. The robust control of the phase transition between the metastable crystalline VO_2_(B) phase and the PIA-VO_2_(B) amorphous phase, via compression and low-temperature annealing, reveals the structure switching behaviour within a tetravalent vanadium-based material for the first time. This highlights exploration of thermodynamically hindered phase transformations with pressure and temperature tuning. Further investigations on the physical mechanism behind the phenomenon are expected, including orbital, electron and magnetic interactions.

## Methods

### Sample preparation

VO_2_(B) nanosheets were synthesized from high pure V_2_O_5_ raw material via a hydrothermal route, with citric acid as the reducing agent. Briefly, 0.182 g V_2_O_5_ powder (1 mmol) and 0.288 g citric acid (1.5 mmol) were added into 20 ml distilled water and continuously stirred to obtain the precursor. The precursor was then transferred into a Teflon-lined autoclave (25 ml capacity, 80% filling). The autoclave was heated to 200 °C at a rate of 3 °C min^−1^ and maintained at 200 °C for 5 h, followed by air cooling to room temperature by switching the power off. The resulting precipitates were washed with deionized water and dried at 80 °C overnight. Transmission electron microscopy (TEM) techniques were applied to check the morphology of the as-synthesized nanosheets at ambient pressure.

### *In situ* high-pressure characterizations

A symmetric DAC was employed to generate high pressure. A stainless steel gasket was pre-indented to a 40 μm thickness, followed by laser-drilling the central part to form a 120 μm diameter hole to serve as the sample chamber. Pre-compressed VO_2_(B) powder pellets and a small ruby ball were loaded in the chamber. Helium or neon was used as the pressure-transmitting medium and the pressures were determined by the ruby fluorescence method^39^. The *in situ* high-pressure angular-dispersive XRD experiments were carried out at the 16BM-D station of the High-Pressure Collaborative Access Team (HPCAT) at the Advanced Phonon Source (APS), Argonne National Laboratory (ANL). A focused monochromatic X-ray beam about 5 μm in diameter (FWHM) and with wavelengths of 0.4246 Å was used for the diffraction experiments. The diffraction data were recorded by a MAR345 image plate and processed with the Fit2D programme. High-pressure infrared spectroscopic experiments were performed at the U2A beamline of National Synchrotron Light Source (NSLS), Brookhaven National Laboratory and the mid-IR beamline of the Canadian Light Source. The infrared spectra were collected in transmission mode by a Bruker FTIR spectrometer using a nitrogen-cooled mid-band MCT (MCT-A) detector. The recorded frequencies were in the range of 600–8,000 cm^−1^ with a resolution of 4 cm^−1^. High-pressure Raman spectra were measured by a Raman spectrometer with a 532.1 nm excitation laser at HPCAT.

For the *ex situ* annealing experiments, the PIA-VO_2_(B) samples were removed with the gaskets after depressurization. They were then annealed at different temperatures (200, 250, 300, 350, 400, 450, 500, 550 °C) each for 5 min within a vacuum furnace, and the resulting phases were checked with synchrotron XRD. While in the *in situ* annealing experiments, the bulky PIA-VO_2_(B) sample was made using a large volume press apparatus and sealed in a glass tube. Then XRD patterns were taken at 30, 60, 90, 120, 160, 190 and 220 °C with electric heating, respectively. The holding time at each temperature was 5 min. Structure refinements were performed with the FULLPROF programme^40^.

### Resistance and magnetic susceptibility measurements

*In situ* electrical resistance was measured by a four-probe resistance measurement system consisting of a Keithley 6,221 current source, a 2182A nanovoltmeter and a 7,001 voltage/current switch system. A DAC device was used to generate pressures up to 30 GPa, and a cubic boron nitride layer was inserted between the steel gasket and diamond anvil to provide electrical insulation between the electrical leads and gasket. Four gold wires were arranged to contact the sample in the chamber for resistance measurements. For the magnetism measurement, 0.1 g sample was made using a large volume press apparatus at 20 GPa for 1 h at room temperature. The DC magnetic susceptibility was measured using a SQUID magnetometer (Quantum Design) with an applied magnetic field of 500 Oe.

### DFT calculations

Total energy and pressure–volume equations of state for the four VO_2_ crystalline phases were calculated at the atomic level using the first-principles plane-wave pseudopotential method^41^ based on the density functional theory, with the CASTEP package^38^. The exchange-correlation function is described by the local density approximation^42^. The ion-electron interactions were modelled with ultrasoft pseudopotentials^43^ for all constituent elements, where the V 4*s*^2^3*d*^3^ and O 2*s*^2^2*p*^4^ electrons were treated as the valence electrons, respectively. The kinetic energy cut-off of 380 eV and Monkhorst-Pack *k*-point meshes^44^ spanning <0.04 Å^−3^ in the Brillouin zone were chosen. The starting structure models were obtained from the Inorganic Crystal Structure Database (ICSD) for VO_2_(A), VO_2_(B), VO_2_(M) and VO_2_(R). The cell parameters and atomic positions within the unit cell of these four phases, under hydrostatic pressure ranging between 0 and 100 GPa (with the interval of 5 GPa), were fully optimized using the quasi-Newton method^45^. The convergence thresholds between the optimization cycles for energy change, maximum force, maximum stress, and maximum displacement were set as 5.0 × 10^–6^ eV per atom, 0.01 eV Å^−1^, 0.02 GPa and 5.0 × 10^–4^ Å, respectively. The optimization terminates when all of these criteria are satisfied. All these computational parameters were tested to ensure the sufficient accuracy for the present purposes.

### Data availability

The data that support the findings of this study are available from the corresponding author upon request.

## Additional information

**How to cite this article:** Wang, Y. *et al*. Reversible switching between pressure-induced amorphization and thermal-driven recrystallization in VO_2_(B) nanosheets. *Nat. Commun.* 7:12214 doi: 10.1038/ncomms12214 (2016).

## Supplementary Material

Supplementary Information Supplementary Figures 1-6
