# Supplementary material for: Reversible switching between pressure-induced amorphization and thermal-driven recrystallization in VO2(B) nanosheets
Source: Nat Commun. 2016 Jul 18;7:12214. doi: 10.1038/ncomms12214 (PMC4960317; doi:10.1038/ncomms12214)
Supplement: Supplementary Information — Supplementary Figures 1-6 [file ncomms12214-s1.pdf]

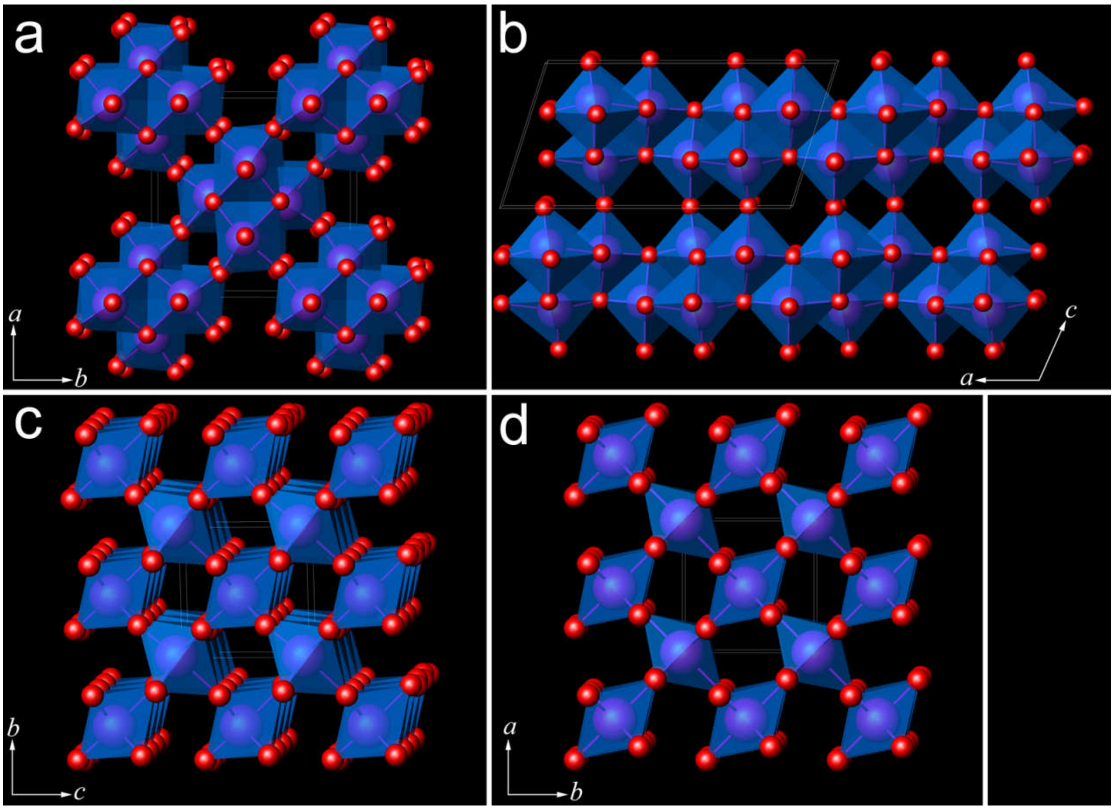

Supplementary Figure 1. Atomic view of the four structure types of VO_2: (**a**) VO_2(A). (**b**) VO_2(B). (**c**) VO_2(M). (**d**) VO_2(R).

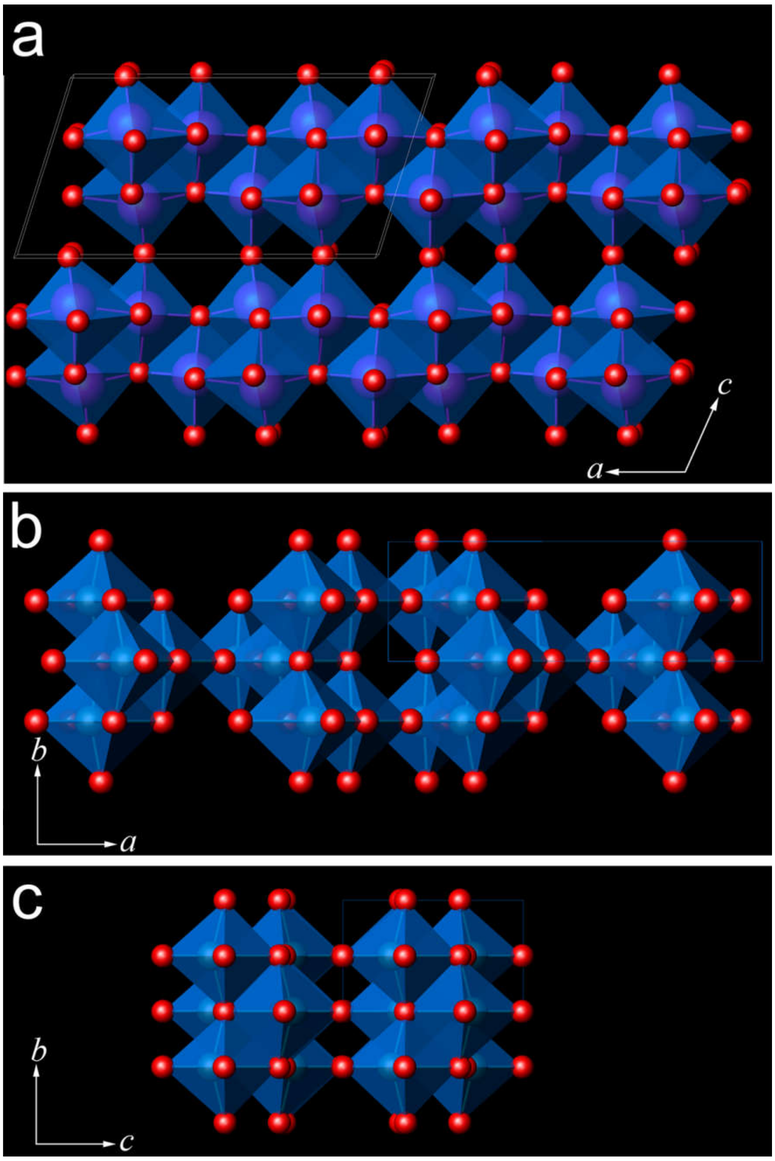

Supplementary Figure 2. (**a**) Crystal structure of VO_2(B) viewed along *b*-axis. (**b**) Crystal structure of VO_2(B) viewed along *c*-axis. (**c**) Crystal structure of VO_2(B) viewed along *a*-axis.

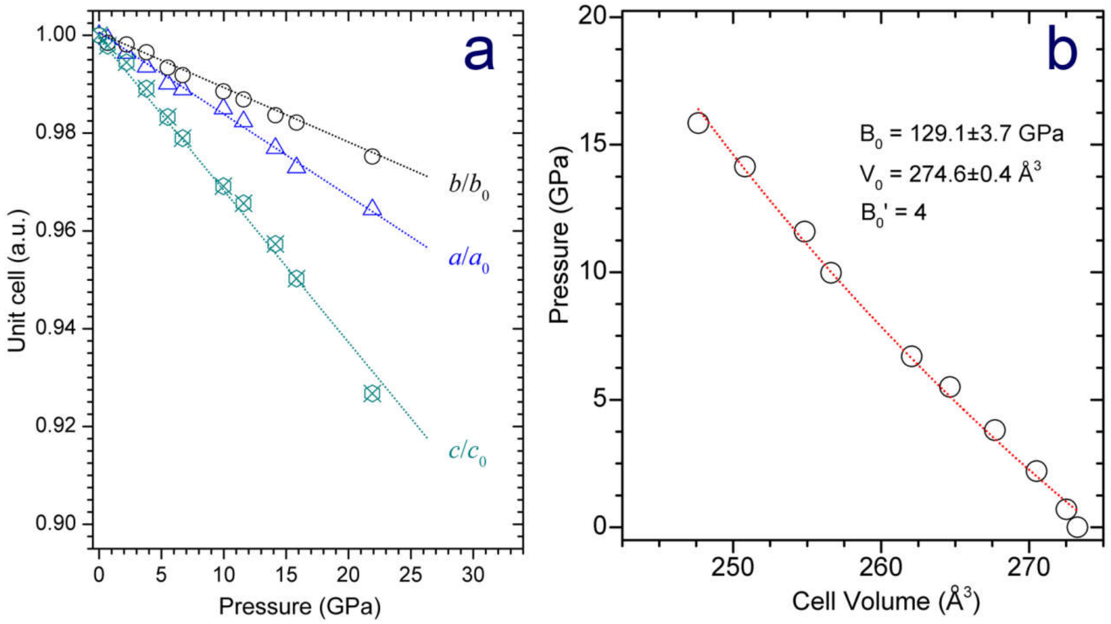

Supplementary Figure 3. (**a**) The lattice parameters of VO_2(B) nanosheets under compression. (**b**) The fitting of the data with the Birch-Murnaghan equation of state (EOS): $P(V) = 3B_0/2 [(V_0/V)^{7/3} - (V_0/V)^{5/3}] \{1 + 3/4(B' - 4) [(V_0/V)^{2/3} - 1]\}$.

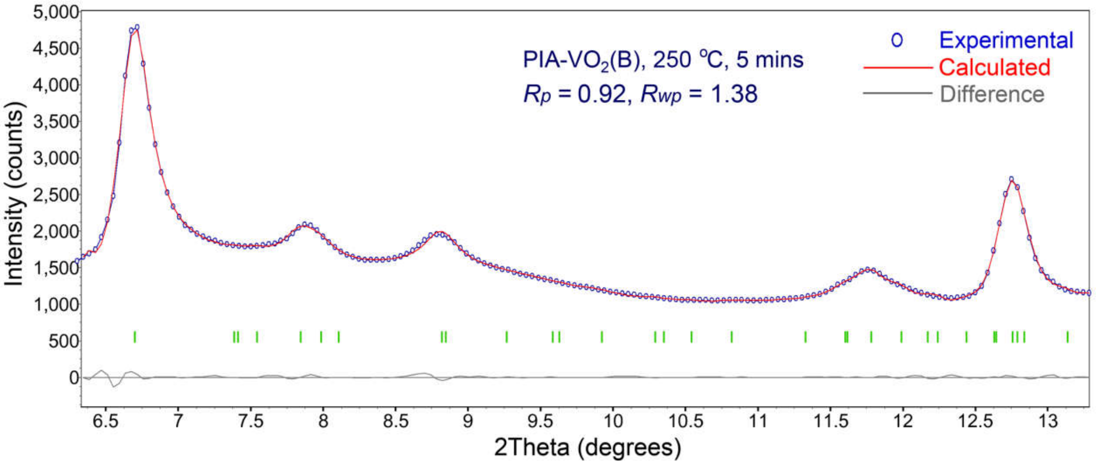

Supplementary Figure 4. Reitveld refinement plot of PIA-VO_2(B) after being annealed at 250 ^{o}C for 5 minutes.

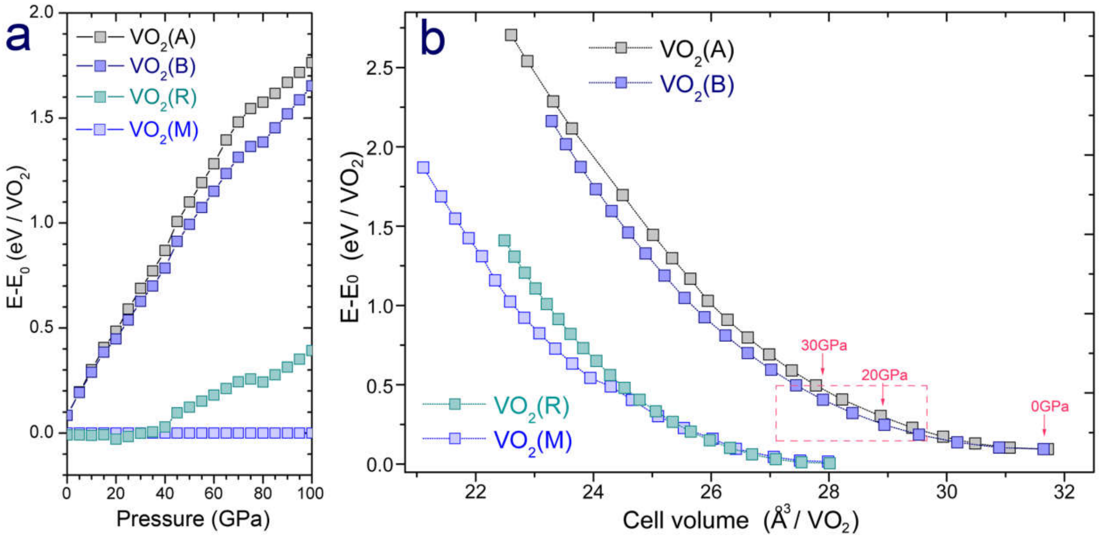

Supplementary Figure 5. Energy evolution of VO_2(A), VO_2(B), VO_2(M) and VO_2(R): (**a**) As a function of pressure, (**b**) as a function of pressure cell volume.

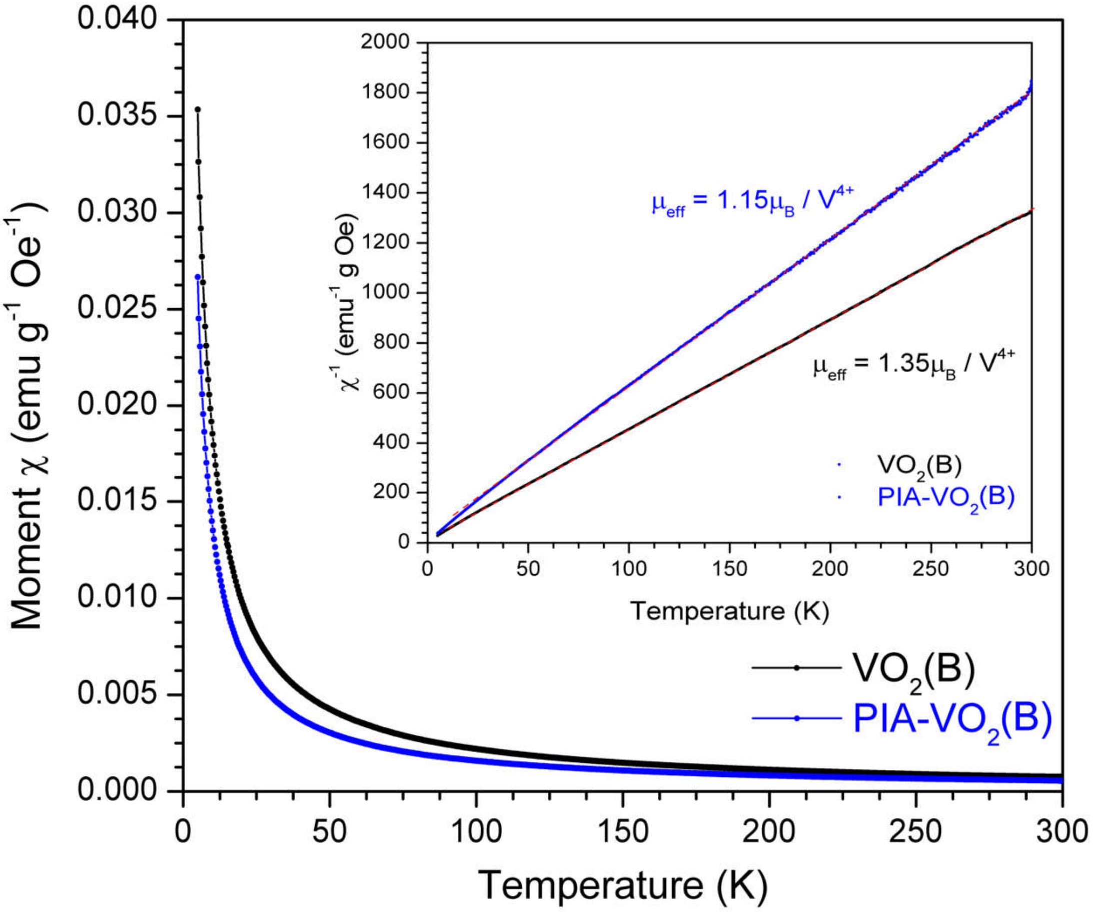

Supplementary Figure 6. Magnetic susceptibility of VO_2(B) and PIA-VO_2(B) as a function of temperature. Applied field: 500 Oe. Insert shows the inverse magnetic susceptibility versus temperature and derived efficient magnetic moment of V^{4+}.
